# Prevention Effect of Allopurinol on Post-Endoscopic Retrograde Cholangiopancreatography Pancreatitis: A Meta-Analysis of Prospective Randomized Controlled Trials

**DOI:** 10.1371/journal.pone.0107350

**Published:** 2014-09-09

**Authors:** Wei-Li Cao, Wen-Shan Yan, Xiao-Hui Xiang, Kai Chen, Shi-Hai Xia

**Affiliations:** 1 Postgraduate Training Base, Affiliated Hospital of Logistics University of the Chinese People's Armed Police Forces, Liaoning Medical University, Jinzhou, China; 2 Department of Hepatopancreatobiliary and Splenic Medicine, Affiliated Hospital of Logistics University of the Chinese People's Armed Police Forces, Tianjin, China; 3 Department of Endemic Area of Orthopedic Center Section III, Affiliated Hospital of Logistics University of the Chinese People's Armed Police Forces, Tianjin, China; University of Valencia, Spain

## Abstract

**Background:**

Pancreatitis is the most common complication of endoscopic retrograde cholangiopancreatography (ERCP) which can be severe and cause death in approximately 10% of cases. Up to now, six randomized controlled trials (RCTs) have been found relevant to the effect of allopurinol on prevention of Post-ERCP pancreatitis (PEP). However, these results remained controversial.

**Objective:**

To conduct a meta-analysis with RCTs published in full text to determine the effectiveness of prophylactic allopurinol of different dosages and administration time in the incidence and severity of PEP.

**Methods:**

Literature search was performed in PubMed, Embase, Web of Science and Cochrane Library from databases inception to May 2014. RCTs comparing the effect of allopurinol with placebo on prevention of PEP were included. Statistical heterogeneity was quantitatively evaluated byχ^2^ test with the significance set P<0.10 or I^2^>50%.

**Results:**

Six RCTs consisting of 1974 participants were eventually included. The incidences of PEP in allopurinol group and placebo group were 8.4%(83/986) and 9.9%(98/988) respectively. Meta-analysis showed no evident prevention effect of allopurinol on the incidence of PEP (RR 0.75, 95%CI 0.39–1.42) with significant heterogeneity (I^2^ = 70.4%, P = 0.005). When studies were stratified according to the dosages and administration time of allopurinol they applied, there was still no evident prevention effect of allopurinol on mild, moderate or severe PEP. However, statistically substantial heterogeneity was presented in the subgroup of moderate PEP when the effect of high dose of allopurinol was analyzed (I_moderate_^2^ = 82.3%, P_moderate_ = 0.018). Statistically significant heterogeneity was also observed in subgroup of mild PEP, when the effect of long adminstration time of allopurinol was investigated (I_mild_^2^ = 62.8%, P_mild_ = 0.068).

**Conclusion:**

The prophylactic use of allopurinol in different dosages and administration time had no effect in preventing incidence and severity of PEP.

## Introduction

Pancreatitis is the most common complication of endoscopic retrograde cholangiopancreatography (ERCP) with incidence being 3.5% in nonselected patients which presents as mild or moderate severity in roughly 90% of patients. However, it is severe and can cause death in approximately 10% of cases [1].

The mechanism of post-ERCP pancreatitis (PEP) remains unclear. Several studies show that free radicals play a great role in the pathogenesis of PEP. Oxygen radicals can lead to capillary endothelial injury, inducing the occurrence of acute pancreatitis [2], [3], [4], [5]. Some free-radical scavengers (superoxide dismutase, catalase), protease inhibitors, and xanthine oxidase inhibitor have been investigated to prevent the frequency of PEP [2], [4], [6], [7]. Allopurinol, a structural analog of the natural purine base hypoxanthine, is capable of inhibiting xanthine oxidase which can catalyze the transformation of hypoxanthine to xanthine and result in the production of oxygen-derived free radical [8]. So allopurinol may play a part in the prevention of PEP through the reduction of oxygen-derived free radical. Many studies in animal models have indicated that the degree of pancreatic inflammation and serum hyperamylasemia was decreased after pretreatment with allopurinol in pancreatography-induced pancreatitis [2], [9].

In clinical trials, six randomized controlled trials (RCTs) have been published in full text about the effect of allopurinol on the prevention of PEP up to now [10], [11], [12], [13], [14], [15]. These results remained controversial. Four prospective studies [10], [11], [13], [15] have yielded negative results while another two studies [12], [14] demonstrated that allopurinol could result in the alleviation of PEP. Katsinelos et al. [12] have indicated that the frequency of PEP was decreased after pretreatment with high-dose of allopurinol. Martinez-Torres et al. [14] have presented the result that the incidences of pancreatitis and hyperamylasemia were decreased after pretreated with allopurinol in patients under high-risk procedures. Two meta-analyses [16], [17] published in 2008 displayed the same results that allopurinol was ineffective for the reduction of PEP. However, a few limits could be observed in previous meta-analyses such as lack of updated RCTs published within recent six years, less studies included and no stratification in terms of allopurinol dosage or adminstration time. Consequently, it is necessary to make a more comprehensive and latest meta-analysis which consists of all RCTs to estimate the effect of allopurinol on the PEP reduction.

The aim of this study is to determine the effectiveness of prophylactic allopurinol of different dosages and adminstration time in the incidence and severity of PEP in RCTs.

## Materials and Methods

### Literature and search strategy

Two reviewers cooperatively searched the following electronic databases: PubMed, Embase, Web of Science and Cochrane Library from databases inception to May 2014. The following related items were searched: allopurinol, placebo, post-endoscopic retrograde cholangiopancreatography pancreatitis, post-ERCP pancreatitis, PEP, randomized controlled trials. Searching strategy was constructed by combining the above items with “AND” or “OR”. No restriction was applied to language. We had also screened reference lists of retrieved articles.

### Study selection criteria

Two reviewers independently assessed the retrieved citations to determine whether they met the inclusion criteria. All disagreements were resolved by discussion with a third reviewer. To be included, the following criteria should be met: 1) patients were scheduled to undergo diagnostic or therapeutic ERCP; 2) random allocation of treatment; 3) the use of allopurinol in intervention group and placebo in control group. Exclusion criteria were: 1) Quasi-randomized clinical trials, retrospective studies, cohort studies and case-control studies; 2) other intervention instead of allopurinol 3) patients with the following characteristics: (i) current pancreatitis, hyperamylasemia, neutropenia, renal dysfunction, decompensated cirrhosis, pregnancy or lactation; (ii) use of anticoagulants, non-steroidal anti-inflammatory drugs (NSAIDS), platelet anti-aggregants or drugs with an interaction with allopurinol; (iii) allergic to allopurinol; (iv) platelet count< 60 ×10^9^/L; (v) unable to swallow or absorb oral medication; (vi) previous sphincterotomy.

### Data extraction and quality assessment

Two reviewers independently conducted data extraction and study quality assessment. All discrepancies encountered were settled eventually by discussion until consensuses were reached. Data that extracted from the included studies consisted of study location, study design, number of patients in each group, inclusion and exclusion criteria of participants, intervention type, dosage of allopurinol used, administration time of allpurinol and PEP patients in different severity degrees. Quality of study was evaluated in six domains comprising sequence generation, allocation concealment, participants' blinding, assessors' blinding, incomplete data, selective reporting and other bias. Each study could be classified as unclear, low risk or high risk of bias for each domain on the ground of Cochrane Handbook 5.1.0 [18].

### Statistical analysis

Statistical analyses were performed with the use of procedure STATA 12.0. Effect was presented by RR with 95%CI for dichotomous variables. Statistical heterogeneity was quantitatively evaluated by χ^2^ test with the significance set P<0.10 or I^2^>50%. Random effect model was used to analyze outcomes which presented significant heterogeneity among studies while fixed effect model to analyze those with non-significant heterogeneity. Publication bias was detected by funnel plot and Egger's test (P<0.05 indicated a significant publication bias).

## Results

### Literature search

**Figure 1:**
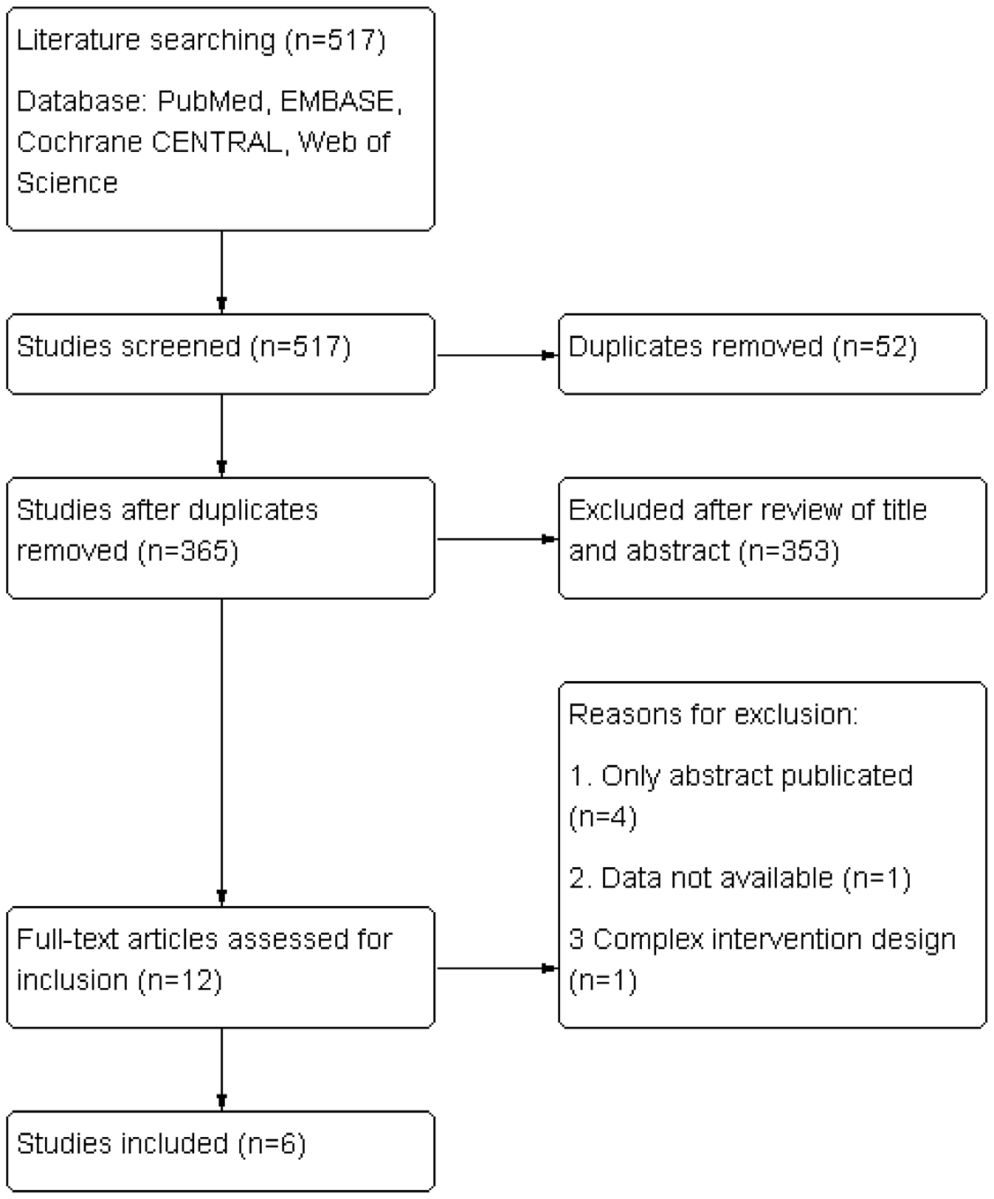
Flow chart of literature selection.

The selection process of study was displayed in Fig. 1. Totally, 517 citations were searched online. After removing the duplicate, title and abstract screening and full text review, 6 RCTs were eventually met the inclusion criteria. No satisfied study was identified by examining the references of the six RCTs. Of all the participants from six RCTs, 986 were treated with allopurinol and 988 with placebo. The characteristics of studies and numbers of patients in different stages were presented in Table 1 and Table 2.

**Table 1:** Characteristics of the included studies.

Reference	Country	Study design	No. patients	Patient inclusion creteria	Patient exclusion creteria	Allopurinol usage and dosage	Outcomes
Abbasinazari 2011	Iran	RCT	29/45	Patients who were to undergo diagnositic or therapeutic ERCP	1) any type of renal failure, 2) any type of anemia, 3) acute pancreatitis during 2 weeks before ERCP, 4) age lower than 20, 5) pregnancy, 6) patients under treatment with azathioprin, 7) refusal or inability to give informed consent.	300 mg at 3 h and 300 mg just before doing ERCP	Amylase concentration; abdominal pain; incidence of PEP
Martinez-Torres 2009	Mexico	RCT	85/85	Patients who were to undergo diagnositic or therapeutic ERCP	1) current pancreatitis or hyperamylasemia 2) non-steroidal anti-inflammatory drugs (NSAIDS) 3) failed ERCP in 12 months 4) previous endoscopic or surgical sphincterotomy 5)use of anticoagulants or platelet anti-aggregants 6) allergic to allopurinol 7) hemoglobin <8 g/dL, platelet count< 60 ×10^9^/L. 8) neutropenia; renal dysfunction; decompensated cirrhosis;9) known or suspected pregnancy or lactation;10) current or recent use of allopurinol or drugs with an interaction with allopurinol, 11) inability to swallow or absorb oral medication	300 mg at 15 h and 300 mg at 3 h before ERCP	Incidence of hyperamylasemia and PEP; ERCP morbidity
Romagnuolo 2008	Canada	RCT	293/293	Patients who were to undergo diagnositic or therapeutic ERCP	1) Haemoglobin level <8 g/dL; 2) platelet count of <60×10^9^/ L; 3) relative neutropenia; significant renal dysfunction; decompensated cirrhosis; 4) allergic to allopurinol; 5) a known or suspected pregnancy or lactation; 6)current or recent use of allopurinol or drugs with a known interaction with allopurinol; 7) an inability to swallow or absorb oral medication; 8)recent acute pancreatitis	300 mg at 1 h before ERCP	proportion of PEP; proportion of patients with local complications of or the need for surgery
Katsinelos 2005	Greece	RCT	125/118	Patients who were to undergo diagnositic or therapeutic ERCP	1) acute pancreatitis ;2) age less than 18 years;3) history of allergy to allopurinol, 4) acute myocardial infarction with in 3 months before ERCP, 5) other severe systemic disease, 6) pregnancy or lactation, 7) refusal to participate.	600 mg at 15 h and 3 h before ERCP	Postprocedur e complica tions; incidence of PEP
Mosler 2005	United States	RCT	355/346	Patients who were to undergo diagnositic or therapeutic ERCP	1) less than 18 years; 2) intrauterine pregnancy; 3) mental disability; 4) incarceration in prison ; 5) active pancreatitis before the procedure; 6) allergy to allopurinol ; 7) actual treatment with allopurinol; 8) contrast allergy; 9) use of mercaptopurine, cyclosporine, chlorpromazine, dicumarol, azathioprine, ampicillin, amoxicillin, or thiazide diuretics; 10) impaired renal function 11) nursing mothers. 12)unable to undergo randomization within 4 hours of the procedure	600 mg at 4 h and 300 mg at 1 h before ERCP	Incidence of PEP
Budzynska 2001	Poland	RCT	99/101	Patients who were to undergo diagnositic or therapeutic ERCP	1) active acute pancreatitis,2) age under 18,3) severe systematic disease, 4) pregnancy or breast feeding, 5) contraindications to corticosteroid administration	200 mg at 15 h and 3 h before ERCP	Incidence of PEP; complications

**Table 2:** Number of patients in different stages.

Author	Patients in allopurinol group	PEP in allopurinol group	PEP classified by severity			Patients in placebo group	PEP in placebo group	PEP classified by severity		
			Mild	Moderate	Severe			Mild	Moderate	Severe
Abbasinazari 2011	29	3	2	1	0	45	5	3	2	0
Martinez-Torres 2009	85	2	2	0	0	85	8	8	0	0
Romagnuolo 2008	293	16	8	6	2	293	12	4	6	2
Katsinelos 2005	125	4	4	0	0	118	21	8	11	2
Mosler 2005	355	46	28	16	2	346	42	24	16	2
Budzynska 2001	99	12	9	2	1	101	8	5	3	0

### Quality of the included studies

**Figure 2:**
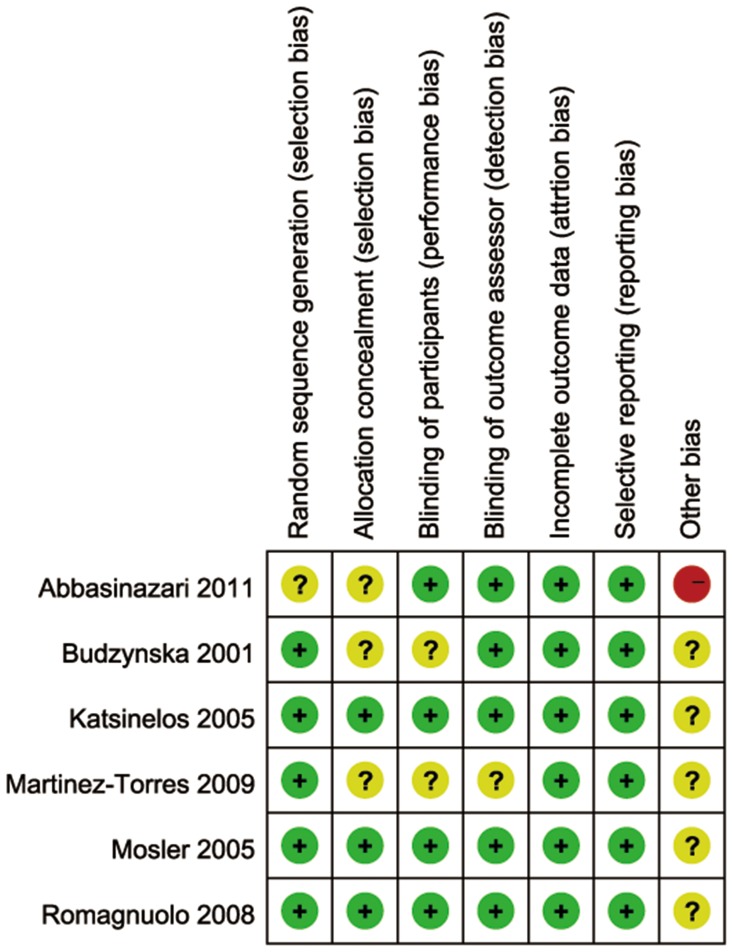
Risk of bias summary.

**Figure 3:**
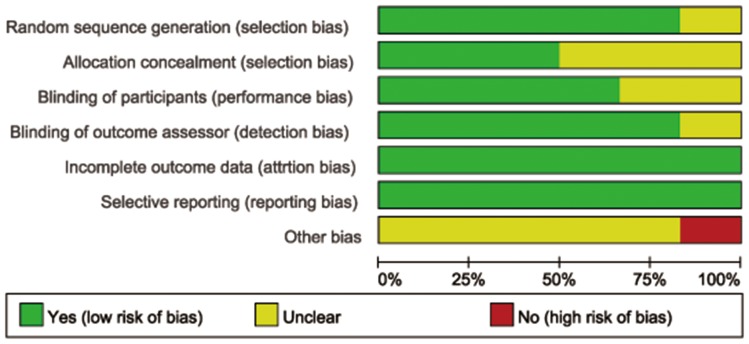
Risk of bias graph.

Among the six included trials [10], [11], [12], [13], [14], [15], sequence generation which means a low risk of bias was clearly conducted and introduced in five studies [10], [11], [12], [13], [14]. Only one study [15] didn't describe the exact method of randomization in spite of simple description in title. Allocation concealment was done and described in three studies [11], [12], [13] with means like a blinded fashion by pharmacy staff, concealed envelopes and coded packets. The remaining three studies [10], [14], [15] shared no information about this domain. With regarding to participants' blinding, four trials [11], [12], [13], [15] presented a low risk for they had illustrated and performed the blinding concretely, whereas in the remaining two trials [10], [14], it remained unclear. In terms of outcome assessors' blinding, five studies [10], [11], [12], [13], [15] shared low risk and only one study [14] presented unclear for this domain. After carefully examining, all the six studies [10], [11], [12], [13], [14], [15] shared low risk of bias regarding to incomplete outcome data. Similarly, all studies reported outcomes they planed previously, suggesting a low risk of bias. No other apparent bias was found among the included studies. Fig. 2 and Fig. 3 show the risk of bias summary.

### Meta-analysis of allopurinol in prevention of developing PEP

**Figure 4:**
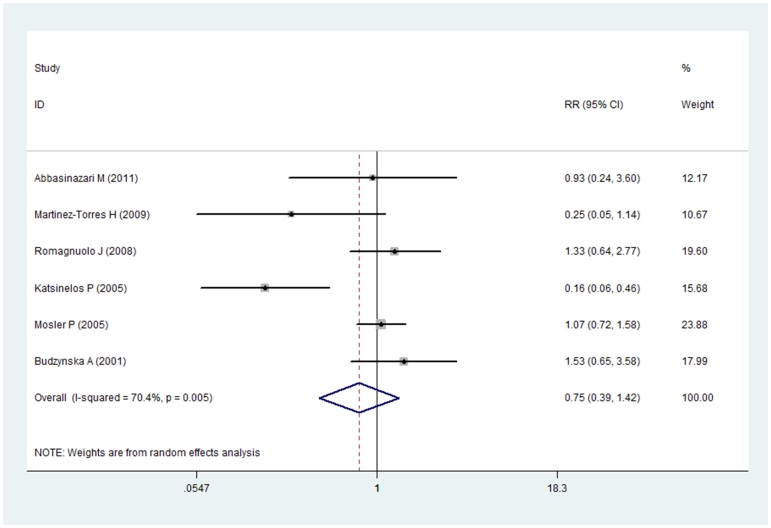
Prevention effect of allopurinol on PEP.

Totally, six RCTs including 1974 participants were included in this meta-analysis. The PEP rates in allopurinol group and placebo group were 8.4% (83/986) and 9.9% (98/988) respectively. Pooled analysis showed no evident prevention effect of allopurinol on the frequency of PEP (RR 0.75, 95%CI 0.39–1.42) with significant heterogeneity (I^2^ = 70.4%, P = 0.005) and therefore random effect model was adopted (Fig. 4).

However, different dosages and administration time of allopurinol were applied in different studies and PEP could be classified as mild, moderate and severe as well. To identify whether allopurinol in various dosages and administration time could present different prevention effect on the severity of PEP, meta-analyses were performed respectively according to different dosages and administration time of allopurinol. (high moderate or low dose, long or short administration time with agreement of two reviewers).

### Results of meta-analysis

#### Low dose of allopurinol

**Figure 5:**
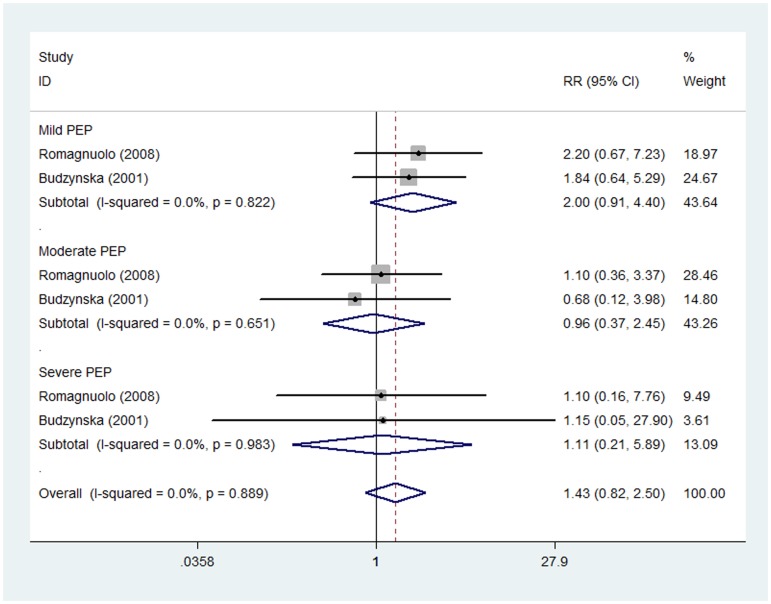
Prevention effect of low dose of allopurinol on PEP in different severity degrees.

Two studies [10], [13] investigated the prevention effect of low dose of allopurinol on the frequency of PEP, including 392 patients treated with allopurinol and 394 with placebo. Pooled analysis showed no significant prevention effect of allopurinol on mild (RR 2.00, 95%CI 0.91–4.40), moderate (RR 0.96, 95%CI 0.37–2.45) or severe PEP (RR 1.11, 95%CI 0.21–5.89) with no statistically substantial heterogeneity in any of the subgroups (I_mild_^2^ = 0.0%, P_mild_ = 0.822, I_moderate_^2^ = 0.0%, P_moderate_ = 0.651, and I_severe_^2^ = 0.0%, P_severe_ = 0.983) (Fig. 5).

#### Moderate dose of allopurinol

**Figure 6:**
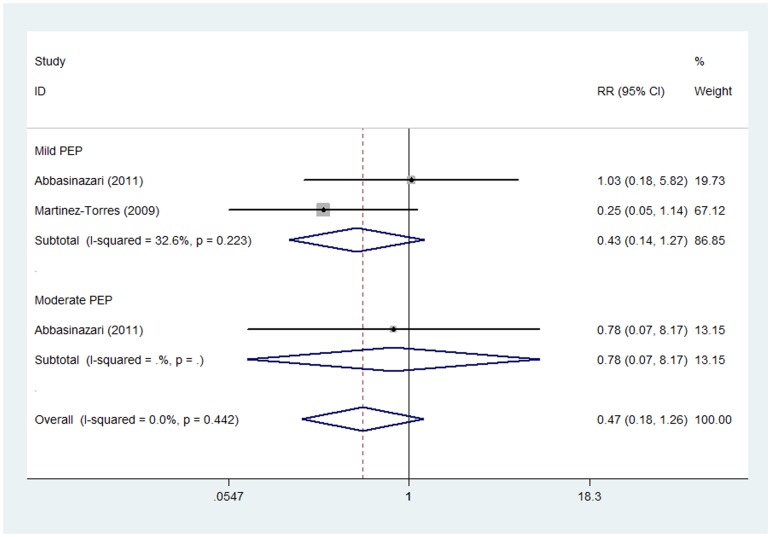
Prevention effect of moderate dose of allopurinol on PEP in different severity degrees.

The prevention effect of moderate dose of allopurinol on the severity of PEP was reported in two studies [14], [15] which consisted of 114 patients treated with allopurinol and 130 with placebo. Subgroup analysis indicated no evident effect of allopurinol on mild (RR 0.43, 95%CI 0.14–1.27) or moderate PEP (RR 0.78, 95%CI 0.07–8.17) (Fig. 6). Notably, no severe PEP case was identified in either of the two trials. Fixed effect model was adopted due to non-significant heterogeneity of two studies (I_mild_^2^ = 32.6%, P_mild_ = 0.223).

#### High dose of allopurinol

**Figure 7:**
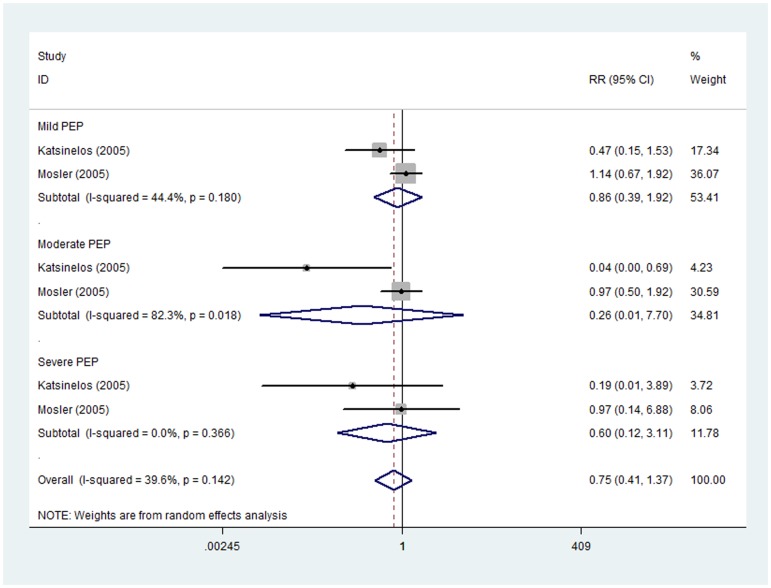
Prevention effect of high dose of allopurinol on PEP in different severity degrees.

Two trials [11], [12], comprising 480 patients in allopurinol group and 464 patients in placebo group, reported the prevention effect of high dose of allopurinol on PEP in different severity degrees. Subgroup analysis showed no substantial prevention effect of high dose of allopurinol on mild (RR 0.86, 95%CI 0.39–1.92), moderate (RR 0.26, 95%CI 0.01–7.70) or severe PEP (RR 0.60, 95%CI 0.12–3.11). Non-significant heterogeneity was observed in either mild PEP subgroup (I^2^ = 44.4%, P = 0.180) or severe PEP subgroup (I^2^ = 0.0%, P = 0.366). However, statistically evident heterogeneity was presented in subgroup of moderate PEP (I^2^ = 82.3%, P = 0.018) and therefore random effect model was applied (Fig. 7).

#### Long administration time of allopurinol

**Figure 8:**
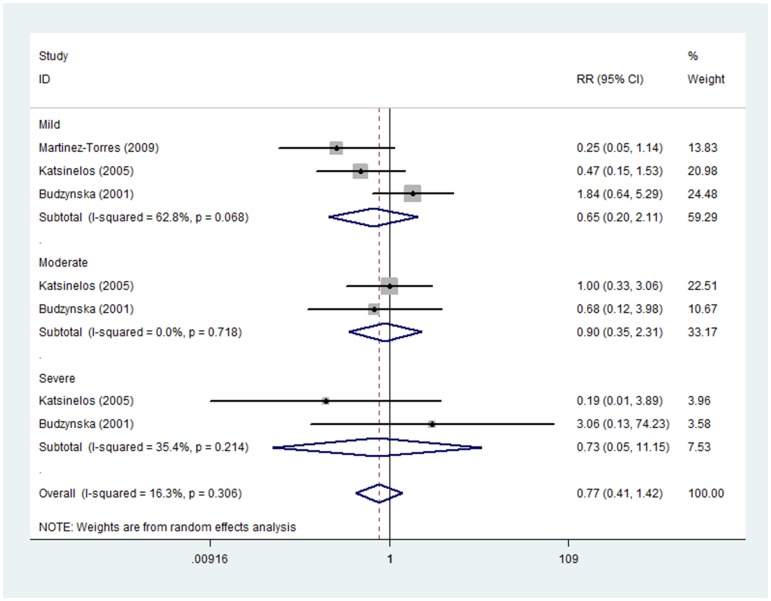
Prevention effect of long adminstration time of allopurinol on PEP in different severity degrees.

Three studies, including 309 patients treated with allopurinol and 304 with placebo, applied long administration time of allopurinol on the incidence of PEP. Pooled analysis showed no evident effect of allopurinol on mild (RR 0.65, 95%CI 0.20–2.11), moderate (RR 0.90, 95%CI 0.35–2.31) or severe PEP (RR 0.73, 95%CI 0.05–11.15). Non-significant heterogeneity was investigated in either moderate PEP subgroup (I_moderate_^2^ = 0.0%, P_moderate_ = 0.718) or severe PEP subgroup (I_severe_^2^ = 35.4%, P_severe_ = 0.214). However, statistically significant heterogeneity was observed in subgroup of mild PEP (I_mild_^2^ = 62.8%, P_mild_ = 0.068) and therefore random effect model was used (Fig. 8).

#### Short administration time of allopurinol

**Figure 9:**
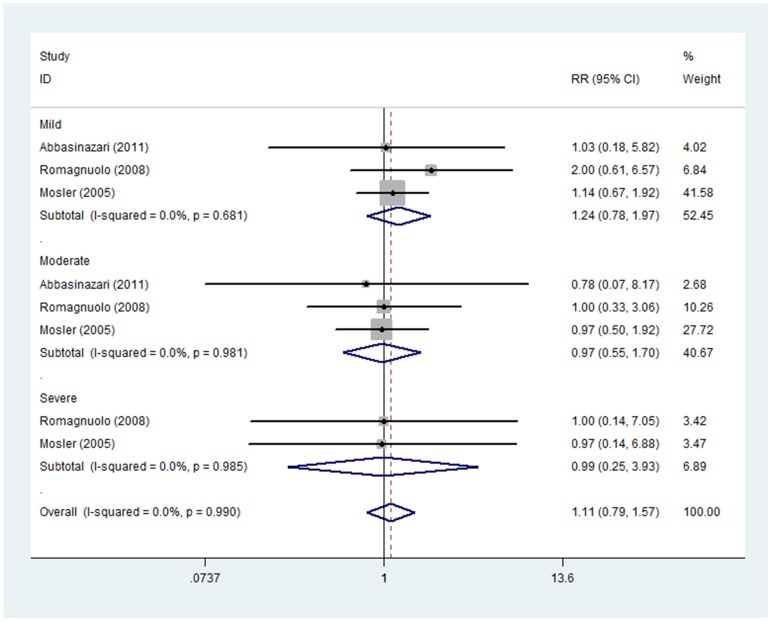
Prevention effect of short adminstration time of allopurinol on PEP in different severity degrees.

The prevention effect of short administration time of allopurinol on the severity of PEP was reported in three studies including 677 patients treated with allopurinol and 684 with placebo. Subgroup analysis indicated no evident effect of allopurinol on mild (RR 1.24, 95%CI 0.78–1.97), moderate (RR 0.97, 95%CI 0.55–1.70) or severe PEP (RR 0.99, 95%CI 0.25–3.93). Fixed effect model was adopted due to non-significant heterogeneity among three studies (I_mild_^2^ = 0.0%, P_mild_ = 0.681; I_moderate_^2^ = 0.0%, P_moderate_ = 0.981; I_severe_^2^ = 0.0%, P_severe_ = 0.985) (Fig. 9).

#### Publication bias

**Figure 10:**
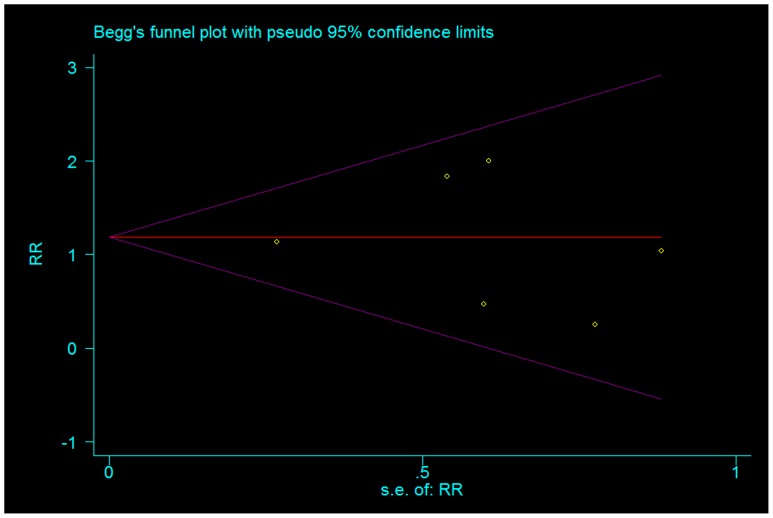
Funnel plot of the included studies assessed the effect of allopurinol on PEP.

Funnel plot and Egger's test were performed to identify potential publication bias. Total prevention effect of allopurinol on PEP evaluated from the six included studies was used as index for funnel plot which presented symmetrical (Fig. 10). Egger's test indicated non-significant publication bias (P = 0.457).

## Discussion

Meta-analysis of the six included RCTs [10], [11], [12], [13], [14], [15] indicated that no significant prevention effect of allopurinol on the frequency of PEP. When studies were stratified according to the dosage of allopurinol, there was still no statistically evident prevention effect of allopurinol on mild, moderate and severe PEP. Five doses of allopurinol were applied in the trials (300 mg, 400 mg, 600 mg, 900 mg and 1200 mg) which had been divided into three levels: low (300 mg, 400 mg), moderate (600 mg) and high (900 mg and 1200 mg) after two reviewers' discussion and agreement.

Two RCTs [10], [13] applied a low dosage of allopurinol. Budzynska et al. [10] showed that allopurinol did not play a significant role in the incidence and severity of PEP. Similarly, Romagnuolo et al. [13] concluded that the overall risk of PEP did not decrease after pretreated with allopurinol. However, it might have potential benefit in high-risk group but potential harm (PEP rates: allopurinol 5.4% vs. placebo 1.5%) in non–high-risk group. The mechanism with regard to this harm is unclear and it could be owing to an idiosyncratic reaction to this medicine [13], nevertheless, no evidence was observed to prove this presumption. Romagnuolo et al. [13] found that the percentage of patients with pancreatic duct injections was significantly higher in allopurinol group(allopurinol 129 vs. placebo 102, P = 0.02), which might result in higher occurrence of PEP in non–high-risk subgroup.

Two studies [14], [15] investigated the prevention effect of moderate dosage of allopurinol. Martinez-Torres et al. [14] indicated that pretreatment with allopurinol decreased the incidences of hyperamylasemia and PEP in patients under high-risk procedures. However, Abbasinazari et al. [15] drew the opposite conclusion with the same dose of allopurinol that there was no difference between allopurinol and placebo for the occurrence of PEP (P = 0.97). According to our analysis, moderate dosage of allopurinol did not have any influence on the prevention of PEP. However, difference could be found in the administration time of the two RCTs. In Martinez-Torres' research, subjects were administrated with allopurinol at 15 h and 3 h before ERCP, while at 3 h and just before doing ERCP in Abbasinazari' study. It is necessary to assess whether administration time plays a part in the effect of allopurinol.

Two trials [11], [12], both published in 2005, applied a high dose of allopurinol in research. Mosler et al. [11] reached the result that the overall frequency of pancreatitis was 12.55%. (allopurinol 12.96% vs. placebo 12.14%; P = 0.52). Besides, there was also no significant difference in mild (allopurinol 7.9% vs. placebo 6.9%), moderate (allopurinol 4.5% vs. placebo 4.6%) or severe (allopurinol 0.6% vs. placebo 0.6%) PEP. On the contrary, Katsinelos et al. [12] held the view that the risk of PEP decreased with the highest dosage(1200 mg) of allopurinol. Administration time of the two studies was not the same, as well. Patients were administrated with allopurinol at 15 h and 3 h before ERCP in Katsinelos' study, while at 4 h and 1 h before ERCP in Mosler' work. So next we tried to investigate whether the effect of allopurinol could be influenced by administration time.

Allopurinol, to our knowledge, can be absorbed approximately 90% in the gastrointestinal tract. It has a rapid onset and 70% of which can transform into a long-lasting active metabolite oxypurinol in liver. Peak plasma levels of allopurinol and oxypurinol can be observed at 1.5 hours and 4.5 hours, respectively. The half life of allopurinol is 1 to 2 hours and that of oxypurinol is about 15 hours [19], [20]. To identify whether the prevention effect of allopurinol could be influenced by administration time, we classified the administration time into two levels: long (15 h and 3 h before ERCP) and short (4 h and 1 h before ERCP, 3 h and just before ERCP, 1 h before ERCP) after two reviewers discussion and agreement.

Long administration time of allopurinol was applied in three RCTs [10], [12], [14]. No evident effect of allopurinol can be observed on mild, moderate or severe PEP in Fig. 8. The metabolite of allopurinol (oxypurinol) was mainly examined in the plasma at 15 h and 3 h before ERCP. Administration of allopurinol at the two time points was long enough to ensure the role of allopurinol for PEP, however, there was no effect of allopurinol observed. We concluded that long administration time of allopurinol played no role in the prevention of PEP. In the three RCTs [10], [12], [14], Budzynska et al. [10] held the view that allopurinol had no effect in preventing PEP, while Katsinelos and Martinez-Torres et al. [12], [14] insisted that allopurinol played a significant role in reducing the incidence of PEP. Katsinelos et al. [12] indicated that their patients group could be considered as a low risk group owing to a small number of patients with sphincter of Oddi dysfunction or previous acute pancreatitis and no pancreatic duct manipulations needed. Martinez-Torres et al. [14] concluded that allopurinol decreased the incidences of PEP in patients under high-risk procedures. The patients were classified into low-risk and high-risk subgroups. Precut sphincterotomy, pancreatic duct manipulation and multiple procedures were considered as risk factors for PEP by Martinez-Torres [14]. Although the results of these two studies by Katsinelos and Martinez-Torres [12], [14] were positive, some differences could be examined. Allopurinol was effective in low risk group of Katsinelos' study [12], while it was effective in high-risk group of Martinez-Torres' study [14]. Therefore, we speculated that it might be the risk factors that affected the results. However, the risk factors were inconsistent with each other among the studies, what is more, the PEP data about risk factors were too limited to analyze whether risk factors affected the role of allopurinol.

Three trials [11], [13], [15] reported short administration time of allopurinol. Subgroup analysis indicated no statistically evident effect of allopurinol on mild, moderate or severe PEP. In Mosler' study [11], allopurinol was administrated at 4 h and 1 h before ERCP. At this time, allopurinol and oxypurinol were both presented with high levels in the plasma. However, Mosler et al. [11] drew the conclusion that allopurinol was ineffective in the prevention of PEP. Romagnuolo et al. [13] using the lowest dose (300 mg) of allopurinol at 1 h before ERCP held the view that allopurinol did not appear to decrease the overall risk of PEP; however, it might be beneficial in high-risk group but potential harm in non–high-risk group. It seems that administration at 1 h before ERCP was just enough to guarantee the work of allopurinol. Abbasinazari et al. [15] indicated that allopurinol did not exert function in occurrence of PEP. In their study, there were total 74 patients undergoing ERCP of which 29 in allopurinol group and 45 in placebo group and therefore the result might be affected by the small number of patients.

Yet there were still some limitations in the above RCTs [10], [11], [12], [13], [14], [15]. Firstly, Katsinelos et al. [12] reported relatively high incidence of PEP in the placebo group (17.8%) which might affect the positive result. This could be attributed to the following aspects: high rate of pancreatic-duct opacification (72.9%), biliary sphincterotomy (73.7%) and pre-cut sphincterotomy (14.4%); varieties of criteria used to define pancreatitis; the comprehensive follow-up [12]. Secondly, the risk factors were inconsistent in above RCTs [10], [11], [12], [13], [14], [15]. For example, male gender, days of hospitalization and administration of allopurinol were considered as risk factors in Katsinelos'study [12], while previous PEP, pancreatic injection and pancreatic therapy were predictors of PEP compared with non-significant risk factors such as sex, number of pancreatic injections, biliary sphincterotomy and pancreatic stent placement in Romagnuolo's study [13]. Thirdly, two trials [14], [15] reported the outcome about amylase, however, we could not analyze it, because Martinez-Torres [14] et al. showed the number of patients with hyperamylasemia, whereas Abbasinazari et al. [15] displayed amylase concentration. Fourthly, it was worth noting that Abbasinazari et al. [15] distinguished the effect between allopurinol and oxypurinol, we considered it unnecessary to make this distinction before making sure the prevention effect of allopurinol.

In terms of the above problems, we recommended the following: in the first place, it was suggested that risk factors in all future RCTs should be classified into patient-related risk factors and procedure-related risk factors. Definite patient-related risk factors (suspected SOD, female gender and previous pancreatitis) and definite procedure-related risk factors (precut sphincterotomy and pancreatic injection) were listed in the table in European guideline which could act as a guide to the future research [21]. In the next place, the reported form related to amylase should remain the same in future study.

Procedural and pharmacological prophylaxes were usually used to prevent the frequency of PEP. The procedural interventions such as guide-wire cannulation [22] and pancreatic stent placement [23] were beneficial in high-risk group. However, pancreatic stenting required a skilled endoscopist and it could act as a high risk of PEP once failed [24]. As for pharmacological prophylaxis, it was possible that non-steroidal anti-inflammatory drugs (NSAIDs) might be useful [25]. Using 100 mg of diclofenac or indomethacin administered rectally would be effective in preventing the incidence of PEP [21]. Two promising agents for decreasing the frequency of PEP,gabexate mesilate and somatostatin, had some problems such as the long time infusion and the cost-effectiveness, particularly in outpatients [26].

A perfect agent should be safe for patients, well tolerated, relatively affordable and have a short administration time and therefore allopurinol seems to be a good choice. It was a safe and useful agent to treat gout and tumor-lysis syndrome and for the reduction of complications such as myocardial infarction, postoperative arrhythmias and mortality after cardiovascular surgery [27]. However, our meta-analysis did not show any evident effect of allopurinol in different dosages and adminstration time on mild to severe PEP. Additionally, an adverse event of allopurinol was reported recently. A 46-year-old man was treated with allopurinol for asymptomatic hyperuricemia. However, pancreatitis and the allopurinol hypersensitivity syndrome which characterized by rash, fever, and internal organ involvement occurred in the patient [28]. Consequently, investigators should be cautious and further examine the role of allopurinol in the prevention of PEP. Although our result was similar to the previous meta-analysis published in 2008 [16], [17], it was more systematic, comprehensive and novel. Additionally, there was no publication bias.

In conclusion, this meta-analysis showed that the prophylactic use of allopurinol in different dosages and administration time had no effect in preventing incidence and severity of PEP. Further well-designed placebo-controlled RCTs are warranted to confirm the effect of allopurinol in preventing PEP.

## Supporting Information

Checklist S1 PRISMA Checklist.
(DOC)
